# Rapid Pathway Evolution Facilitated by Horizontal Gene Transfers across Prokaryotic Lineages

**DOI:** 10.1371/journal.pgen.1000402

**Published:** 2009-03-06

**Authors:** Wataru Iwasaki, Toshihisa Takagi

**Affiliations:** 1 Department of Computational Biology, University of Tokyo, Kashiwa, Chiba, Japan; 2 Database Center for Life Science, Research Organization of Information and Systems, Bunkyo-ku, Tokyo, Japan; Université Paris Descartes, INSERM U571, France

## Abstract

The evolutionary history of biological pathways is of general interest, especially in this post-genomic era, because it may provide clues for understanding how complex systems encoded on genomes have been organized. To explain how pathways can evolve *de novo*, some noteworthy models have been proposed. However, direct reconstruction of pathway evolutionary history both on a genomic scale and at the depth of the tree of life has suffered from artificial effects in estimating the gene content of ancestral species. Recently, we developed an algorithm that effectively reconstructs gene-content evolution without these artificial effects, and we applied it to this problem. The carefully reconstructed history, which was based on the metabolic pathways of 160 prokaryotic species, confirmed that pathways have grown beyond the random acquisition of individual genes. Pathway acquisition took place quickly, probably eliminating the difficulty in holding genes during the course of the pathway evolution. This rapid evolution was due to massive horizontal gene transfers as gene groups, some of which were possibly operon transfers, which would convey existing pathways but not be able to generate novel pathways. To this end, we analyzed how these pathways originally appeared and found that the original acquisition of pathways occurred more contemporaneously than expected across different phylogenetic clades. As a possible model to explain this observation, we propose that novel pathway evolution may be facilitated by bidirectional horizontal gene transfers in prokaryotic communities. Such a model would complement existing pathway evolution models.

## Author Summary

Many biological functions, from energy metabolism to antibiotic resistance, are carried out by biological pathways that require a number of cooperatively functioning genes. Hence, underlying mechanisms in the evolution of biological pathways are of particular interest. However, compared to the evolution of individual genes, which has been well studied, the evolution of biological pathways is far less understood. In this study, we used the abundant genome sequences available today and a novel algorithm we recently developed to trace the evolutionary history of prokaryotic metabolic pathways and to analyze how these pathways emerged. We found that the pathways have experienced significantly rapid acquisition, which would play a key role in eliminating the difficulty in holding genes during the course of pathway evolution. In addition, the emergence of novel pathways was suggested to have occurred more contemporaneously than expected across different phylogenetic clades. Based on these observations, we propose that novel pathway evolution can be facilitated by bidirectional horizontal gene transfers in prokaryotic communities. This simple model may approach the question of how biological pathways requiring a number of cooperatively functioning genes can be obtained and are the core event within the evolution of biological pathways in prokaryotes.

## Introduction

The evolution of biological pathways has attracted increasing attention in recent years [1]–[3]. Since this research area originated more than 60 years ago [4], several models have been proposed for the evolutionary mechanisms of biological pathways, to explain the building principles of biological systems (e.g., [5]). Recently, the advance in genome sequencing technologies and the development of novel computational tools have enabled researchers to study pathway evolution (i.e., the evolution of pathways) in a comprehensive and systematic manner.

Unlike the evolution of individual genes, the evolution of genes that function cooperatively (e.g., genes constituting biological pathways) cannot be understood intuitively in many cases. As genetics has shown, biological pathways sometimes lose their positive effects on the survival of the host species when some parts of their component genes are absent from genomes. This would suggest that, to acquire a novel biological pathway by inventing its genes individually, a genome might have to retain those genes until it completes all components of the pathway, a process that would be evolutionarily disadvantageous [4].

To date, several noteworthy models of pathway evolution have been proposed. Recent studies have shown that the patchwork model, in which enzymes from different pathways are recruited and combined to generate a novel pathway [5], could play a major role in pathway acquisition events, compared to other models (e.g., the pathway duplication, enzyme specialization, and retro-evolution models; for a review, see [3]). These observations come mainly from sequence similarity analyses between biological pathways [6] and close investigations into individual pathways [7]. Nevertheless, the reconstruction of pathway evolution on a genomic scale and over the tree of life has suffered from several obstacles.

To trace pathway evolution, it is necessary to estimate the pathways of ancestral species. In general, this can be achieved by estimating the ancestors' gene-content vectors, whose elements represent the existence or nonexistence of genes belonging to each ortholog group, and then projecting them onto metabolic databases. An intuitive solution for gene-content estimation uses methods based on maximum-parsimony [8]–[10]. Given a phylogenetic tree and an ortholog table (a matrix comprised of the gene-content vectors of all extant species investigated), these methods estimate the existence or nonexistence of each ortholog group at each branching point on the tree, just as the maximum parsimony method does in molecular phylogenetics. However, the ancestral gene content estimated by using these methods is drastically affected by changing the relative penalties between gene gain and gene loss events [10]. This artificial effect requires iterative experiments with a number of different penalty parameter sets, a process whose complexity has prevented researchers from investigating the unambiguous history of pathway evolution. The substantial difficulty in choosing these penalties arises from the fact that gene gain/loss rates are actually highly variable depending on the biological background of the host species (e.g., massive horizontal gene transfers [HGTs] and parasitization sometimes result in exceptional numbers of gene gains and losses, respectively [11],[12]), which is incompatible with the universal application of one set of gene gain/loss rates.

Recently, we developed an algorithm that can estimate ancestral gene content precisely without an artificial effect by estimating the most likely rates of gene gains and losses over a phylogenetic tree [13]. We applied this algorithm to the present problem and investigated the general tendencies of pathway evolution on the scale of whole genomes and the tree of life. In the present study, we focused on prokaryotic rather than eukaryotic lineages, because prokaryotic and eukaryotic modes of genome evolution are highly divergent and should be treated separately [13]. Metabolic pathways were chosen for analysis because they are the best understood and best described biological pathways in prokaryotes.

## Results

### Reconstruction of Gene-Content Evolutionary History

**Figure 1:**
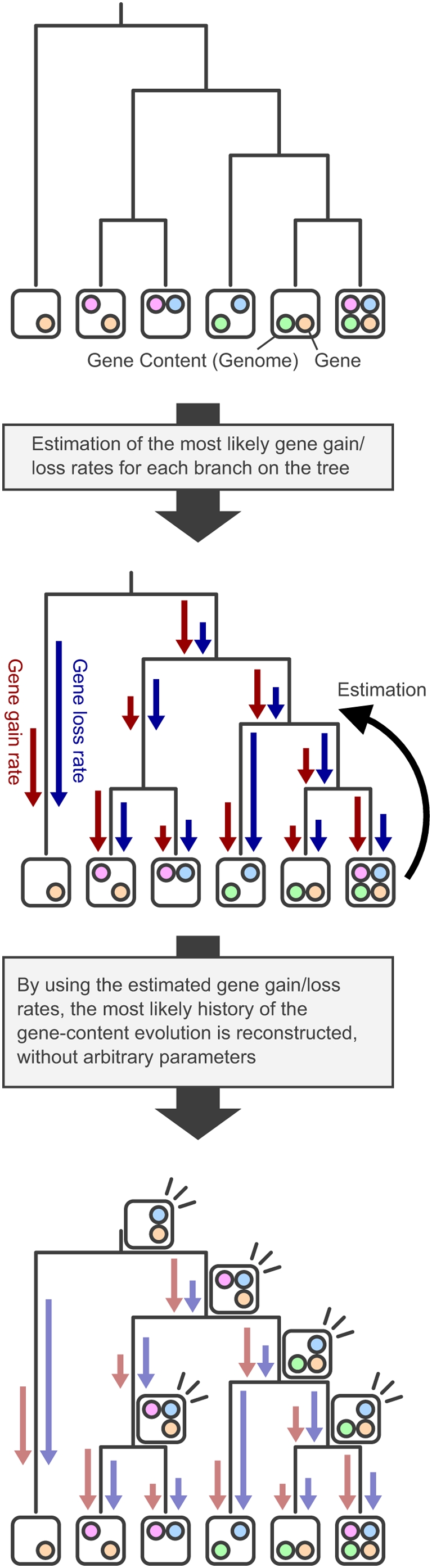
Schematic representation of the gene-content reconstruction algorithm adopted in the present study. In this model example, there are six genomes and four ortholog groups. The algorithm first estimates the most likely gene gain/loss rates on each branch of the phylogenetic tree. Then, it reconstructs the most likely history of the gene-content evolution by using the estimated gene gain/loss rates, without arbitrary parameters.

To reconstruct the gene content of ancestral prokaryotic species, we adopted an algorithm that can effectively estimate gene-content evolutionary history [13]. This algorithm, which is summarized in Figure 1, estimates the gene content of each ancestral species (i.e., each intermediate node on a phylogenetic tree) as an integer vector whose elements represent the numbers of genes for each ortholog group. To deal with the heterogeneous gene gain/loss modes, the algorithm's evolution model can estimate rates of gene gains and losses for each individual branch on a tree automatically without given artificial parameters. The estimated rates are then used to calculate the probabilities of gene gain/loss events over the phylogenetic tree (e.g., the probability of gene gains on a branch with a high gene gain rate becomes large, the reverse occurs with gene losses), and a set of ancestral gene-content vectors with the highest probability is computed.

**Figure 2:**
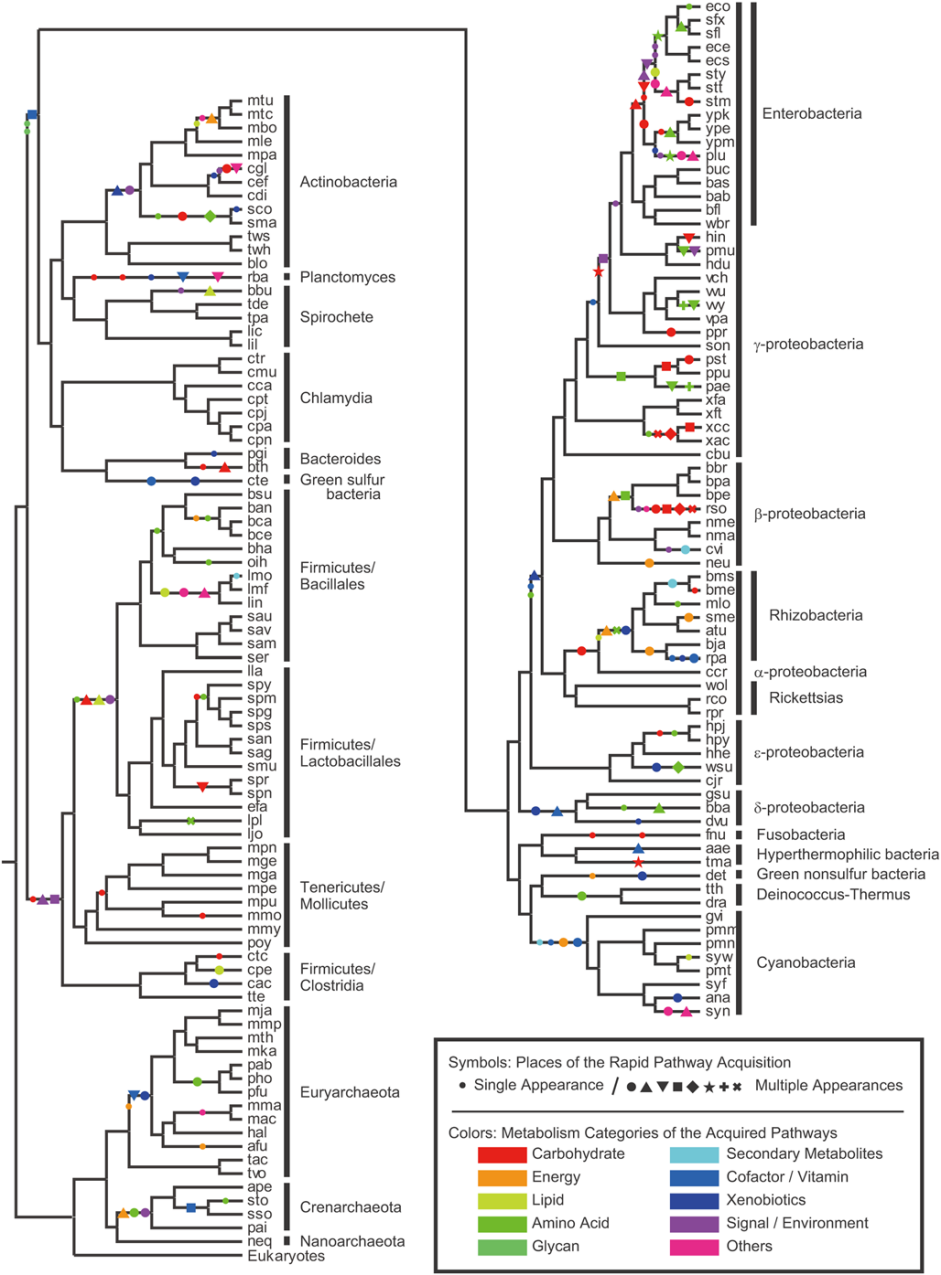
Prokaryotic species analyzed and the phylogenetic tree, overlaid with rapid pathway acquisition events. Species codes and taxonomic classifications are from the KEGG database [15] (for complete species names, see Table S1). Branch lengths reflect evolutionary time estimated by Ciccarelli *et al.* [14] to some extent, without impairing readability. Symbols on the tree indicate the places where pathways were acquired rapidly (see the main text). Small circles indicate a single appearance, whereas large symbols of the same shape and color indicate the independent acquisition of the same pathway at different places on the phylogenetic tree. Colors indicate the metabolic categories in which the pathway is involved; shapes have no special meaning.

This algorithm requires pre-computation of a species phylogenetic tree and an expanded ortholog table that describes numbers of genes instead of the usual existence/nonexistence [13]. We used the phylogenetic tree of life produced by rigorous phylogenetic analysis [14] as the most comprehensive and most resolved tree of life available today, and we used an extended ortholog table derived from the manually annotated KEGG Orthology database [15]. The intersection of the phylogenetic tree and the expanded ortholog table encompassed 142 species of Eubacteria and 18 species of Archaea (Figure 2; for complete names, see Table S1). Using these as the input data set, we reconstructed the most likely gene content of the ancestral prokaryotic species.

Although many of the branches on the phylogenetic tree used in the present study [14] had strong bootstrap supports, some branches had rather weak supports. Hence, for the statistical tests conducted below, we tested whether the observations were robust when we used alternative tree topologies and confirmed that they were supported as well (see Methods).

### Tracing Pathway Evolution on the Tree-of-Life Scale

**Figure 3:**
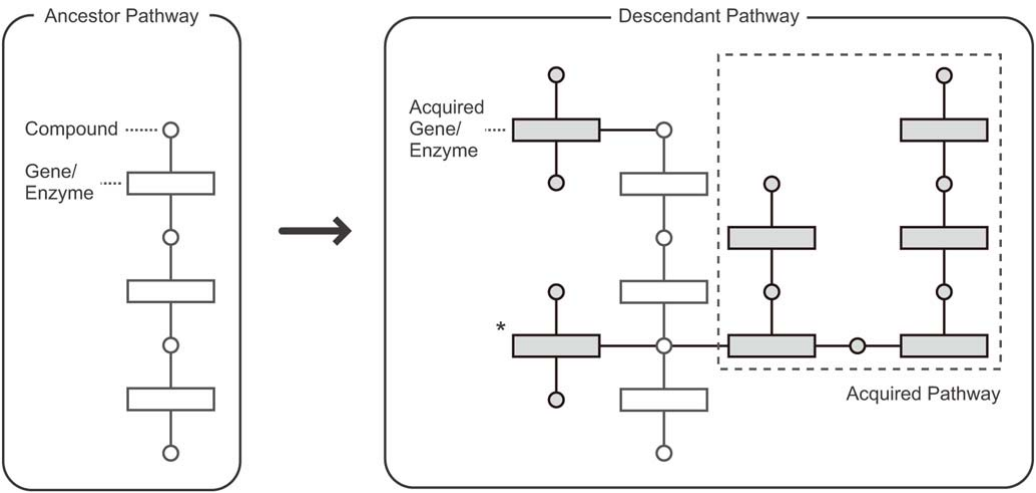
Schematic representation of the procedure used to detect the acquired pathways. Note that the gene/enzyme connected via a compound that already existed in the ancestral metabolic network is not comprised in the acquired pathway (indicated by an asterisk).

To investigate general tendencies of pathway evolution, we traced how metabolic pathways have evolved to date over all prokaryotic lineages (Figure 3). First, the probable reaction catalog of each of the ancestral and extant species was deduced using the KEGG annotations between metabolic reactions and genes (enzymes) that constitute the species gene content. We discarded information on reaction directionalities because our focus was not on orders, but on the cooperativeness of genes. Second, for each pair of an ancestral species and its direct/indirect descendant species on the phylogenetic tree, we subtracted the ancestor's gene content from the descendant's. The resultant genes corresponded to a set of genes acquired during the evolutionary period between the two species. Finally, every gene pair in each of the gene sets acquired together was connected if its catalyzing reactions shared at least one compound that was not contained in the ancestor's reaction catalog, which may have comprised a set of chemical compounds that was already exploited. This procedure not only prevented us from connecting genes that shared universal metabolites only (e.g., H_2_O and ATP) but also let us systematically define which metabolites were trivial to each species (i.e., if a species already metabolized compound X, the acquisition of two genes connected by X would not necessarily mean that these genes function cooperatively; otherwise, they are likely to function in a coordinated manner). Because pathway definitions that connect genes via such trivial or species-specific compounds cause substantial problems in pathway analyses [16], the procedure described here was developed.

We did not require that the ultimate substrates and/or products of the pathways already be represented in the metabolic network of the ancestor, because there would be no *a priori* reason to assume pathways not connected to the existing network do not contribute to the host survival. This is because (1) ultimate substrates can be absorbed from the environment, (2) ultimate products can be useful in contexts other than metabolism (e.g., they can function as signaling molecules), and (3) alternative unknown pathways may connect the newly acquired pathways and the existing networks. We treated the genes/enzymes whose reaction products would be consumed in the consecutive spontaneous reactions according to the KEGG annotation as if they also catalyze the corresponding spontaneous reactions. We adopted this procedure because our focus was not on whether the enzymes directly catalyze the reactions, but on whether the enzymes would function cooperatively. In addition, we allowed genes on the same reaction (e.g., genes constituting multi-subunit enzymes) to be connected, because they should also be the genes that function cooperatively.

In this way, the history of pathway evolution over the tree of life was deduced as a set of graphs whose nodes and edges corresponded to genes and reactions, respectively, at the resolution of phylogenetic tree branches. Hereafter, the terms edges and branches are confined to metabolic reactions and phylogenetic relationships, respectively.

### Pathways Grow Significantly as Genomes Evolve

**Figure 4:**
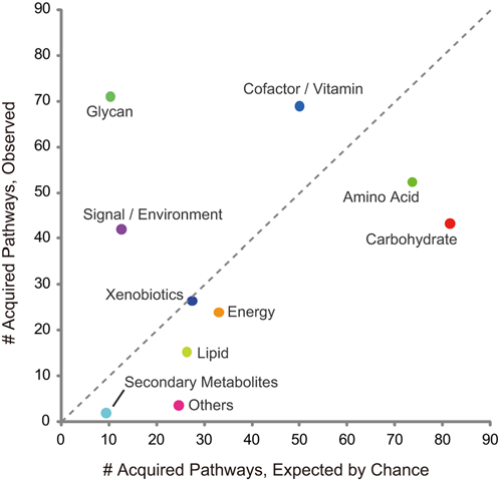
Functional distribution of the acquired pathways. Number of pathway acquisition events for each functional category is shown. The vertical axis corresponds to the observed numbers, whereas the horizontal axis represents the expected numbers based on the functional distribution of all genes in this study. The functional categories above the dashed 45° line were more frequently observed than expected.

Because the subject of our study was pathway evolution, we were interested in sets of genes that function coordinately and appeared together on a genome. Hence, we searched among each of the deduced graphs for ones that contained at least five connected genes. Changing this number in the range of three to eight did not affect the results. Over the entire phylogenetic tree, we extracted 379 such connected graphs by excluding redundancies, and we call these graphs “acquired pathways.” The functional distribution of the acquired pathways is shown in Figure 4; the functional categories above the dashed line (i.e., glycan metabolism, environmental information processing/signaling, and cofactor/vitamin metabolism) were more frequently observed than expected.

Next, we used this comprehensive evolutionary history to examine whether a phenomenon that can be called pathway evolution really exists. That is, if the independent acquisition of genes can also result in a comparable number of acquired pathways, pathway evolution would be anything but a remarkable phenomenon. To test this possibility, we shuffled the relationships between genes and reactions and investigated whether a comparable number of acquired pathways was observed. To avoid bias due to genes not being assigned to the previously identified pathways, we used only genes that were associated with reactions in the data set. In addition, we preserved the original gene gain/loss numbers on each branch to eliminate bias from the heterogeneous distribution of gene gain/loss events (e.g., an exceptional number of gene gains would itself lead to the acquisition of pathways). As a result, there were 31.9±18.9 acquired pathways in the shuffled data sets, which was significantly fewer than the original 379 pathways (*N* = 1000, p<0.05). This indicates that pathways have actually grown beyond the independent acquisition of genes over the history of genome evolution.

### Pathway Evolution Occurs Rapidly, not Gradually

As a general rule, to acquire multiple genes that function cooperatively, evolving genomes might be able to choose from two possible tactics: the gradual acquisition of the genes or their rapid acquisition and retention. As stated earlier, the former scenario might raise the difficulty of keeping genes that have weak effects on host survival, especially in the case of prokaryotic evolution, in which the evolutionary pressure on genome size is very strong and such genes can be promptly discarded from the genomes [4]. Nonetheless, this gradual evolution scenario may also be supported by the fact that even a well-studied genome such as that of *Escherichia coli* contains thousands of non-essential genes [17], suggesting that the existence of genes that have weak effects may be less disadvantageous than generally thought. Therefore, we examined whether the gradual acquisition scenario would hold true.

**Figure 5:**
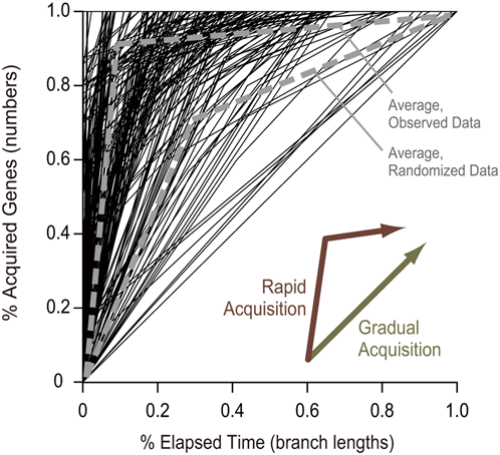
Modes of pathway acquisition. Each sequential line from the bottom left corner to the top right represents how rapidly a pathway was acquired. A segment corresponds to one branch during the evolutionary period when the pathway was acquired. Its slope represents the proportion of acquired genes divided by the branch length derived from the linearized phylogenetic tree (see Methods). Segments were sorted in descending order of their slopes to visualize how strongly the acquisition was biased. If the gradual acquisition scenario holds true, sequential lines will approach the 45° line, whereas the rapid acquisition scenario will produce lines that are strongly convex upward. To permit comparison, representative lines for the present and the randomized data are shown as gray dashed lines.

To examine how pathway acquisition actually took place, we visualized its mode in Figure 5. Its horizontal axis represents relative evolutionary time, and the vertical axis represents the proportion of acquired genes to all genes constituting the pathway being investigated. The history shown seems to support the rapid acquisition scenario, and this observation was statistically supported, as described in the Methods section (p<0.05). This rapid gene gain scenario is consistent with the highly heterogeneous modes of gene-content evolution; that is, genomes change drastically by sometimes expanding or shrinking quickly [13].

### Rapid Pathway Evolution Accompanies HGTs in Groups

The next question is how the rapid acquisition of pathways was achieved. To focus on the rapidly evolved portions of the acquired pathways, we searched for graphs that were acquired within one branch, and found 156 “rapidly acquired pathways” that contained at least three connected genes over the phylogenetic tree (Figure 2, colored symbols). Increasing this cutoff value to four reduced the number to 62, which was not sufficient to conduct the statistical tests described below.

We hypothesized that HGTs contribute to rapid evolution because they play a key role in prokaryotic genome evolution [18]. Probable HGT instances were detected if genes belonging to the same ortholog group (i.e., gene sets that are exclusively similar to each other among all genes in the data set) appeared independently at different places on the phylogenetic tree [10]. In particular, we investigated the independent appearances of *pathways*, which we defined as rapidly acquired pathways that (1) appeared at different places on the tree and (2) shared at least three genes in common.

Among the rapidly acquired pathways, we found that a significant proportion appeared independently several times, as 95 of the 156 rapidly acquired pathways were compiled to give 33 unique pathways (Figure 2, large symbols; those of the same shape and color indicate the independent acquisition of the same pathway at different places on the phylogenetic tree). This value was significantly larger than that expected by chance (p<0.05; see Methods). This suggests that such HGTs, not as individual genes but as gene groups functioning coordinately, promote rapid pathway evolution. Because this observation is consistent with comparative genomics studies of closely related species in which recent pathway acquisition via operon transfers has been detected [19], some of the pathway transfers might be due to horizontal transfers of operons, at least those that occurred relatively recently.

### Original Pathway Acquisition Occurs Contemporaneously across Phylogenetic Clades

Operon transfers might be able to carry existing pathways, but they do not seem to be able to develop novel pathways. Therefore, the next question is how these pathways originally appeared. Because we reconstructed pathway evolution at the depth of the tree of life, it may be possible to shed light on this question. We focused on the oldest acquisition of the 33 repeatedly acquired pathways. Here, the pathway acquisition time periods were estimated by using a linearized tree, which assumes a constant rate of evolution and enables the inference of temporal relationships between evolutionary events on a phylogenetic tree [20]. Note that the following statistical evaluation was confined to bacterial taxa only, because it is quite difficult to assess temporal relationships between bacterial and archaeal taxa using the molecular clock.

We found that, among the repeatedly acquired pathways, the oldest acquisition was soon followed by the second-oldest acquisition. In other words, the first and second acquisitions of a rapidly acquired pathway seem to have occurred more contemporaneously in different phylogenetic clades than expected by chance. The mean difference was 0.13 time units, which was a significantly smaller value than the background. This was confirmed to be neither because of artificial effects in selecting old branches nor because of intrinsic bias in the original pathway-acquiring branches (p<0.05; see Methods). The fact that branch lengths of a linearized tree can be affected by some sort of artifacts is noteworthy. Nonetheless, the present analysis would have been robust to such effects, because observations were based on the comparison against the background time difference calculated by using the same linearized tree, and it is expected that such artifacts would have been canceled out in the comparison.

## Discussion

In this study, we traced and analyzed the evolutionary history of metabolic pathways on prokaryotic genomes at the depth of the tree of life. The reconstruction was conducted carefully, by considering the trivial and species-specific compounds and estimating the most likely gene gain/loss rate for each phylogenetic branch. This process yielded four findings: (1) Pathways have grown beyond the random acquisition of individual genes, (2) obstacles to pathway acquisition would be overcome by the rapid acquisition of genes that would function cooperatively, (3) this rapid evolution was due to massive horizontal transfers as gene groups, and (4) the original acquisition of the pathways seems to have occurred more contemporaneously than expected across different phylogenetic clades.

Importantly, the functional categories that were emphasized in the pathway acquisition events (i.e., glycan metabolism, environmental information processing/signaling, and cofactor/vitamin metabolism) are the categories that are likely to be substantially affected by the environment. Preferable glycan structures would be influenced by cohabiting organisms and the chemical/physical properties of the environment, environmental information processing/signaling should respond to the environment adequately by definition, and the usefulness of cofactor/vitamin metabolism pathways would also be deeply affected by the existence of related molecules in the environment. Thus it would be reasonable to assume that these pathways have been more acquired than other pathways including ones in the central metabolism, by corresponding to changes in the environment.

One possible explanation for the contemporaneous acquisition of pathways might be that some portions of the original pathway acquisition occurred through bidirectional gene transfers instead of unidirectional operon transfers. If this is the case, pathways could evolve in a prokaryotic community and likely be acquired contemporaneously in multiple phylogenies. A possible advantage of this pathway evolution model is that the recruitment of genes from different phylogenies could expand available gene/enzyme space, which would be preferable in quickly developing pathways before the evolutionary pressure discards genes, possibly by complementing the effect of the patchwork model [5]. To our knowledge, this is the first study to propose this pathway evolution model, based on a comprehensive analysis of actual pathway evolutionary history.

As a possible instance of such pathway evolution in prokaryotic communities, we found a pathway for lysine biosynthesis via α-aminoadipate and *N* ^2^-acetyl-l-lysine among the repeatedly acquired pathways estimated in this study. This pathway was estimated to have been acquired three times in the ancient era, in the ancestors of Crenarchaeota, Deinococcus-Thermus, and Pyrococcus (Figure 2, large green circles). It is suggested that there was no proper lysine biogenesis pathway at the last common ancestor of life, and at least five different pathways, including the one above, are believed to have developed after the diversification of life [21],[22]. It is interesting that all three of these phylogenetic clades live in similar hot environments [23], which does not contradict the assumption that this pathway evolved in a prokaryotic community. Another interesting observation is the repeated acquisition of pathways on carbohydrate metabolism among intestinal (e.g., Enterobacteria and Bacteroides) and pathogenic (e.g., Pseudomonas, Xanthomonas, and Ralstonia) bacteria (Figure 2, red symbols). The habitats of these bacteria are characterized by rich source of carbohydrate, and thus it is reasonable that many carbohydrate metabolic pathways have evolved among these phylogenetic clades.

The recent technical advances in environmental genomics are remarkable, and more than 100 metagenomics projects are ongoing. Although many contigs detected in such projects cannot currently be assigned to individual taxa, making it difficult to discuss pathway evolution in each phylogenetic clade by using those data sets, in some studies, most of the environmental genes are binned to individual species [24]. Moreover, this situation might be improved in the future with the use of sequencers that can read longer sequences than can existing sequencers. Above all, metagenomic data have an important advantage in that they can ensure the certain existence of prokaryotic communities. We therefore expect that the emergence of biological pathways in prokaryotic communities might be further studied experimentally and computationally, for example by taking advantage of metagenomics technologies.

## Methods

### Data Set and Reconstruction of Gene-Content Evolutionary History

To prepare the expanded ortholog table, we downloaded the KEGG Orthology data from the KEGG database [15] and counted the numbers of genes for each ortholog group over the whole 160 species. The phylogenetic tree was downloaded from the supplementary website of the paper reporting the tree [14]. To prepare the metabolic reaction set, we downloaded the KEGG Pathway database [15], which contains information on the chemical reactions catalyzed by each KEGG ortholog group.

To reconstruct the gene content of the ancestral prokaryotic species, we adopted a previously developed algorithm that is summarized in Figure 1 and fully described in a separate paper [13]. We converted the ortholog table and the phylogenetic tree into table format in the R language and ran the R implementation of the algorithm. Then, the program reconstructed the most likely gene content of the ancestral species by estimating the most likely gene gain/loss rates on all branches of the phylogenetic tree. The computation took about 20 h on a Linux machine with a 3-GHz Intel Pentium 4 processor.

### Enumeration of Acquired Pathways

To enumerate the acquired pathways, we adopted the following procedure. First, we selected every pair of an ancestral species and its descendant from the phylogenetic tree: The ancestor was always an internal node, and the descendant could be either an internal or an external node. The descendant did not need to be a *direct* descendant (i.e., child) of the ancestor. Second, for every species pair, we counted all the pathways that were acquired through the evolutionary process from the ancestor to the descendant, as described in the main text. Third, to avoid counting the same event redundantly, we excluded any pathway that was a subgraph of another pathway whose acquisition periods covered that of the former.

To enumerate the rapidly acquired pathways, we adopted the following procedure: First, we selected every pair of an ancestral species and its *direct* descendant (i.e., child) from the phylogenetic tree. Then, for every pair, we counted all the pathways that were acquired through the evolutionary process from the ancestor to the descendant, as described in the main text.

For the repeatedly acquired pathways, we counted the rapidly acquired pathways that (1) appeared at different places on the tree and (2) shared at least three genes in common. When genes belonged to the same ortholog group and were shared beyond phylogenetic clades, three possibilities could be considered: multiple geneses, multiple losses, and HGTs. Among these, the second and third possibilities should be more likely, because genes in the same ortholog group are *exclusively* similar to each other among all genes in the data set and thus are likely to have some sort of evolutionary relationship, although the possibility of convergent evolution might remain. Between the second and third possibilities, we exploited the advantage of our algorithm. As described above and in Figure 1, our algorithm can estimate the most likely gene gain/loss rates on each phylogenetic branch, and thus it provides the probabilities of the multiple loss scenario and the HGT scenario based on those gene gain/loss rates. If the probability of the HGT scenario is greater than the probability of the multiple loss scenario, the algorithm will estimate the acquisition of the same ortholog group at difference places on the tree, and the HGT scenario was adopted.

### Statistical Test for the Rapid Acquisition Scenario

Statistical support for the rapid pathway acquisition scenario was obtained by evaluating how the acquisition sequential lines in Figure 5 were displaced from the 45° line. If areas surrounded by the sequential lines and the 45° line were larger than expected by chance, the pathway acquisition could be judged to have occurred rapidly. This indicator was borrowed from the Gini coefficient in economics, which uses the area surrounded by Lorenz curves to measure inequality of wealth distribution.

The mean area was 0.409 for our data, and we examined the significance of this value by shuffling the gene acquisition periods for all ancestral-descendant species pairs where the pathway acquisition occurred. We generated real numbers that were randomly distributed between 0 and the total length of the phylogenetic branches between the two species, and these random numbers were treated as the acquisition periods of each gene constituting the acquired pathways. Then, the mean area of the acquisition sequential lines was calculated for the randomized data in the same way. The experiment was repeated 1,000 times and the expected area was 0.206±0.003, which showed that the pathway acquisition was actually biased to the rapid acquisition scenario with a significance level of 0.05.

### Statistical Test for the HGTs in Groups

Statistical support for the HGTs in groups was obtained as follows. For all 156 rapidly acquired pathways, the same number of genes that existed in the descendant but not in the ancestor were chosen randomly, as in the above test to eliminate the bias originating from species selection. Instances of the independent acquisition of the same three genes at different places on the phylogenetic tree were then counted. The experiment was repeated 1,000 times, and the expected number of such instances was 0.11±0.33, which showed that rapid pathway acquisition accompanied HGTs in groups (p<0.05).

### Linearized Tree and Statistical Test for Contemporaneous Pathway Acquisition

The linearized tree was constructed as described [20] with the branch lengths produced by the maximum likelihood method [14]. The pathway-acquisition periods were expected to be the midpoints of the branches. As described in the main text, the following statistical evaluation was confined to bacterial taxa.

Two statistical tests for contemporaneous pathway evolution were performed. First, to test the possibility that selecting two branches of the oldest and second oldest acquisition simply resulted in temporally close branches, we randomly selected 95 pathways from all of the rapidly acquired pathways and grouped them into 33 groups as in the original grouping. The oldest and second oldest branches were then extracted from each group, and periodical differences between them were calculated. The mean difference was 0.20±0.03 (*N* = 1000), which was significantly larger than 0.13 (p<0.05)

Second, to test the possibility that the original 95 branches were biased to close relationships in themselves, we randomly selected 33 pairs from the 95 branches and investigated their mean differences. The result was 0.17±0.02 (*N* = 1000), which also was significant (p<0.05).

### Alternative Tree Topologies

The alternative tree topologies were constructed as follows. We downloaded the amino acid sequence alignment created by concatenating the orthologous genes that are conserved over the tree of life from the supplementary website of the paper reporting the comprehensive phylogenetic tree [14]. Then we constructed 20 bootstrap alignments and their phylogenetic trees by applying the maximum likelihood method (PhyML, [25]). We conducted the same series of analyses using the 20 alternative phylogenetic trees to test whether the same observations were obtained.

## Supporting Information

Table S1 The 160 prokaryotic species analyzed in this study.
(0.04 MB XLS)
